# Type I Interferons in Systemic Autoimmune Rheumatic Diseases: Pathogenesis, Clinical Features and Treatment Options

**DOI:** 10.31138/mjr.270324.tis

**Published:** 2024-06-30

**Authors:** Konstantinos Drougkas, Charalampos Skarlis, Clio Mavragani

**Affiliations:** 1 Department of Physiology, School of Medicine, National and Kapodistrian University of Athens, Athens, Greece,; 2 Joint Academic Rheumatology Program, National and Kapodistrian University of Athens, Athens, Greece

**Keywords:** type I interferons, systemic autoimmune rheumatic diseases, autoimmunity, systemic lupus erythematosus, Sjögren’s disease, dermatomyositis

## Abstract

Type I interferon (IFN) pathway dysregulation plays a crucial role in the pathogenesis of several systemic autoimmune rheumatic diseases (SARDs), including systemic lupus erythematosus (SLE), Sjögren’s disease (SjD), systemic sclerosis (SSc), dermatomyositis (DM) and rheumatoid arthritis (RA). Genetic and epigenetic alterations have been involved in dysregulated type I IFN responses in systemic autoimmune disorders. Aberrant type I IFN production and secretion have been associated with distinct clinical phenotypes, disease activity, and severity as well as differentiated treatment responses among SARDs. In this review, we provide an overview of the role of type I IFNs in systemic autoimmune diseases including SLE, RA, SjD, SSc, and DM focusing on pathophysiological, clinical, and therapeutical aspects.

## INTRODUCTION

Interferons (IFNs) represent a group of functionally related cytokines of innate immunity displaying antiviral, antimicrobial, antiproliferative, and antitumor activities as well as immunomodulatory effects on both innate and adaptive immune responses.^1^ To date, three distinct types of IFNs are recognised: type I, type II and type III.^2^ Accumulating evidence highlights that dysregulation of the type I IFN pathway represents a main pathogenetic event in several autoimmune conditions, including both organ-specific autoimmune disorders such as autoimmune thyroid and inflammatory bowel disease and systemic autoimmune rheumatic diseases (SARDs), including systemic lupus erythematosus (SLE) and Sjögren’s disease (SjD).^3^

A comprehensive research effort is currently in progress, to explore whether type I IFNs can serve as a marker for distinct clinical and laboratory features, as well as differentiated treatment responses in the context of multiple SARDs.^4^ Furthermore, effective therapeutic agents targeting the type I IFN pathway have been developed for SARDs treatment.^5^ In the present review we aim to summarise the current knowledge and provide an update regarding the implications of the type I IFN axis in the development, clinical manifestations, and treatment response of the key SARDs, incorporating latest research findings.

## OVERVIEW OF TYPE I IFN SYSTEM

**Figure 1.**
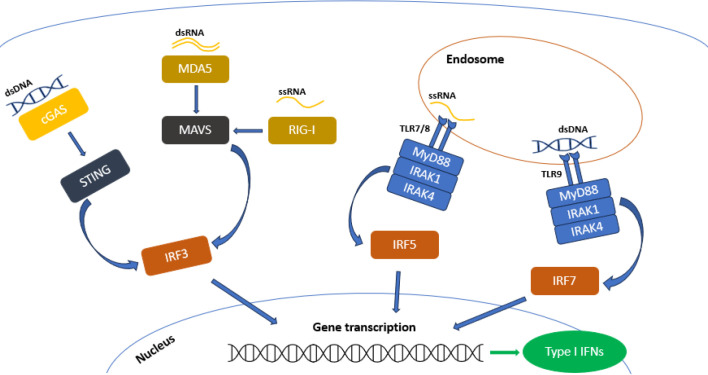
Type I IFN production. Activation of endosomal TLR7/8 by RNA or TLR9 by DNA results in MyD88-dependent phosphorylation and activation of IRF5 and/or IRF7 which induce transcription of type I IFNs. Additionally, activation of cytosolic nucleic acid sensor cGAS by DNA or MDA5 and RIG-I by RNA can activate IRF3 through STING and MAVS respectively, also inducing type I IFN gene transcription. TLR: Toll-like receptor; MyD88: Myeloid differentiation factor 88; IRAK: Interleukin-1 receptor-associated kinase; dsDNA: Double-stranded DNA; ssRNA: Single-stranded RNA; dsRNA: Double-stranded RNA; IRF: Interferon regulatory factor; IFN: Interferon; cGAS: Cyclic guanosine monophosphate–adenosine monophosphate synthase; MDA5: Melanoma differentiation-association protein 5; RIG-I: Retinoic acid-inducible gene 1; STING: Stimulator of interferon genes; MAVS: Mitochondrial antiviral-signaling protein.

Type I IFNs include several subtypes, such as IFNα, IFNβ, IFNδ, IFNω, IFNε, IFNτ, IFNζ, and IFNκ. The IFNα subgroup can be further divided into 13 subtypes, which are encoded by 13 homologous genes situated on the short arm of chromosome 9.^6^ Type I IFNs (primarily IFNα and IFNβ) are potent antiviral cytokines secreted by almost all cell types in response to the detection of microbial products (such as lipopolysaccharide (LPS)) and foreign nucleic acids.^7^ It is widely acknowledged that plasmacytoid dendritic cells (pDCs) are the main producers of IFNα, while various other cell types, including epithelial cells, dendritic cells, phagocytes, fibroblasts, and synoviocytes, secrete IFNβ.^8^ Normally, the production of type I IFNs is triggered when various stimuli are recognised by pattern recognition receptors (PRRs). These various stimuli include microbial products, exogenous pathogens, endogenous self-nucleic acids, apoptotic debris, neutrophil extracellular traps (NETs), and immune complexes (ICs).^9^ PRRs include toll-like receptors (TLRs), found on cell surfaces and within endosomal membranes of the cells responsible for type I IFN production, which can bind to LPS, and DNA or RNA, respectively.^10^ Furthermore, the cytosolic receptors retinoic acid-inducible gene 1 (RIG-I) and melanoma differentiation-association protein 5 (MDA5) are specialised for sensing RNA, while cyclic GMP-AMP synthase (cGAS) is responsible for detecting DNA.^11^ Activation of these receptors leads to the stimulation of IFN stimulatory genes protein (STING) and the production of type I IFNs. In particular, within pDCs, the recognition of nucleic acids by endosomal membrane-bound TLR7/8 or TLR9 triggers a cascade of events. First, it induces activation of myeloid differentiation factor 88 (MyD88), which subsequently interacts with interleukin-1 (IL-1) receptor-associated kinase (IRAK) 1 and IRAK 4, forming a complex. This complex activates the IFN regulatory factor (IRF) 5 and/or IRF7 through phosphorylation, which act as transcription factors. Ultimately, the translocation of IRF5 into the nucleus initiates the transcription of genes encoding type I IFNs, IL-6, tumor necrosis factor (TNF), and IL-12. Simultaneously, IRF7 prompts the production of type I IFNs, especially IFNα (**Figure 1**).^12^

**Figure 2.**
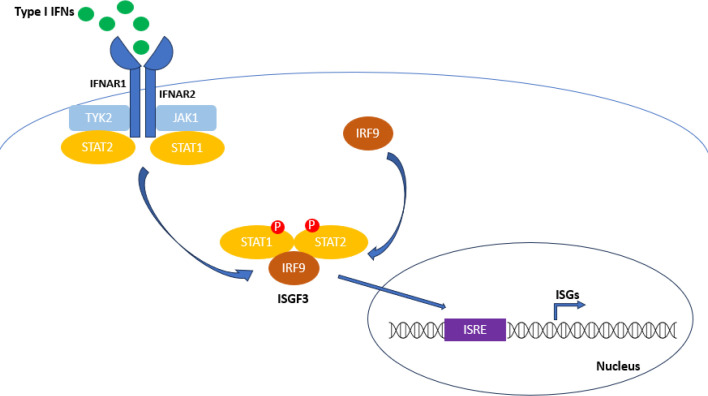
Type I IFN signaling pathway. Type I IFNs bind to a heterodimeric transmembrane receptor composed of the subunits IFNAR1 and IFNAR2. IFNAR1 activates TYK2 while IFNAR2 activates JAK1 and these kinases phosphorylate STAT1 and STAT2 resulting in the dimerization and binding of these molecules to IRF9 to form ISGF3. Upon translocation to the nucleus, ISGF3 initiates the transcription of ISGs through binding to ISREs in DNA. IFN: Interferon; IFNAR: Interferon-α/β receptor; JAK1: Janus kinase 1; TYK2: Tyrosine kinase 2; STAT: Signal transducer and activator of transcription; IRF: Interferon regulatory factor; ISGF3: Interferon-stimulated gene factor 3; ISRE: Interferon-stimulated response elements; ISGs: Interferon-stimulated genes.

IFNα and IFNβ transduce their signal by binding to IFN-α/β receptor (IFNAR), which are present on the cell membrane of most nucleated cells. The interaction between IFNAR and IFNα/β leads to the dimerisation of IFNARs; their subunits IFNAR1 and IFNAR2 individually bind to and activate distinct members of the Janus kinase (JAK) protein families: IFNAR1 activates tyrosine kinase 2 (TYK2), while IFNAR2 activates JAK1.^13^ Subsequently, cytoplasmic transcription factors signal transducer and activator of transcription (STAT) 1 and STAT2 can undergo phosphorylation by JAKs, and IRF9 can bind to STAT1/STAT2 heterodimers, forming the heterotrimeric complex IFN-stimulated gene factor 3 (ISGF3). Upon translocation to the nucleus, ISGF3 is capable of initiating the transcription and the subsequent upregulation of hundreds of IFN-stimulated genes (ISGs) through binding to IFN-stimulated response elements (ISREs) in DNA.^14^ Significantly, IRF7 stimulates the expression of ISGs, which also include IRF7, establishing a positive feedback loop within the type I IFN signaling pathway (**Figure 2**).^15^

## MAJOR TRIGGERS OF TYPE I IFN PRODUCTION IN SYSTEMIC AUTOIMMUNITY

### Genetic factors

Numerous functional gene variants have been recognised as contributors to the production of type I IFNs conferring an elevated risk for the development of autoimmune disorders. For example, the three-prime repair exonuclease 1 (TREX1) gene encoding for the corresponding 3′-5′ DNA exonuclease has been involved in the clearance of aberrant DNA, while TREX1 gene mutations have been associated with numerous diseases characterised by excessive type I IFN activation such as Aicardi-Goutieres syndrome (AGS), SLE and systemic sclerosis (SSc).^16^ In the same context, TLR7 and TLR9 gene variants have been linked to SLE development.^17,18^ Notably, a recent study showed that a novel TLR7 gain-of-function variant (TLR7^Y264H^) can cause human and murine lupus.^19^ Similarly, IRAK1 is involved in the modulation of TLR signaling, and polymorphisms of the IRAK1 gene have been linked to the pathogenesis of SLE.^20^ Next, a missense allele of IFN-induced with helicase C domain 1 (IFIH1) (rs1990760), the gene encoding for MDA5, has been implicated in elevated expression of ISGs in patients with anti-dsDNA (double-stranded DNA) positive SLE.^21^ Furthermore, functional polymorphisms affecting several IRFs have been linked to the development of autoimmune disorders. IRF5 gene variants are associated with SLE, discoid and subacute cutaneous lupus, SSc, and SjD.^22^ Rare and low-frequency missense variants in the interacting proteins B lymphoid tyrosine kinase (BLK) and B cell adaptor protein with ankyrin repeats (BANK1) can impair suppression of IRF5 in human B cell lines and increase pathogenic lymphocytes in murine lupus.^23^ Aside from IRF5, IRF7 risk haplotypes have been described in SLE pathogenesis and progression of fibrosis in SSc.^24,25^ Furthermore, multiple genetic studies have identified IRF8 as a significant risk gene for autoimmune diseases,^26^ while a recent one demonstrated that rs2280381 is likely a causal variant that modulates IRF8 expression.^27^ PTPN22W*, a classical autoimmune gene variant, can increase susceptibility for SjD, especially the low type I IFN subgroup, suggesting the presence of distinct genetic backgrounds between low and high type I IFN SjD subsets.^28^ Lastly, genome-wide association studies (GWAS) have contributed to the identification of susceptibility loci associated with SARDs, implicating genes such as IRF4, IRF5, IRF8, STAT4 in SSc and IRF5, ITGAM, KIAA1542, PXK, FCGR2A, PTPN22, STAT4 in SLE.^29–32^ Interestingly, case-case GWAS comparing SLE patients with high versus low type I IFN activity, have identified novel risk loci including PRKG1, PNP, and ANKS1A, which were not detected with case-control studies.^33,34^

### Nucleic acid-containing immune complexes

Although pDCs readily activate in response to viral antigens, they do not react to naked self-nucleic acids, and the production of type I IFNs is protected both by internalisation of TLR7/9 within the cells and the presence of nucleases in the tissue milieu.^35^ However, ICs containing “self” DNA or RNA, can be shuttled into endosomes and activate TLRs inducing type I IFN production from pDCs.^36^ Notably, impaired clearance of apoptotic cells and extracellular genetic material can provide the necessary antigenic material for the formation of these ICs. This was initially demonstrated by Ronnblom and colleagues in a series of experiments that showed the capacity of ICs containing antigenic material from necrotic and apoptotic cells to induce IFNα production by pDCs.^37,38^ These nucleic acid-containing ICs can be internalised by binding to Fc gamma receptors at the cell surface and shuttled into the endosome to activate TLRs. Specifically, ICs containing DNA, such as those formed by autoantibodies binding to nucleosomes, can activate TLR9. Contrariwise, RNA-containing ICs, formed by autoantibodies complexed with U1 small nuclear RNA in pDCs, can activate TLR7.^35^ In this context, Barrat et al. demonstrated that TLR7/9 oligonucleotide inhibitors significantly decrease IFNα production by pDCs, expanding on the significance of TLR signaling pathways in the setting of systemic autoimmunity.^39^

### Neutrophil extracellular traps

Another mechanism contributing to the induction of type I IFN production by pDCs involves the ability of amphipathic peptides to form complexes with extracellular nucleic acids, facilitating the intracellular transport of this interferonogenic material into endosomes.^36^ Instances of these peptides include the sole member of the human cathelicidin family, LL-37, and the chemokine (C-X-C motif) ligand 4 (CXCL4). Specifically, CXCL4-DNA complexes can significantly enhance TLR9-mediated pDCs activation and subsequent IFNα production in the context of SSc.^40^ LL-37 plays a pivotal role in the stabilisation of ICs generated through NETs. NETosis encompasses a peculiar form of neutrophil cell death, characterised by the formation of NETs, decondensed chromatin threads complexed with cytoplasmic antimicrobial peptides.^41^ SLE-derived NETs externalise significant amounts of LL-37, protecting NET-associated DNA from degradation. Coupled with anti-dsDNA antibodies, these NET-derived ICs of antibody, DNA, and LL-37 are potent inducers of type I IFN production from pDCs.^42^ Notably, it was shown that IFNs, as well as autoantibodies against LL-37, can prime neutrophils for NETosis. Subsequently, these NETs can activate pDCs to produce type I IFNs, therefore creating a self-perpetuating inflammatory cycle that provides additional NETs to sustain type I IFN production.^43^

### Endogenous retroelements and mitochondrial DNA

**Figure 3.**
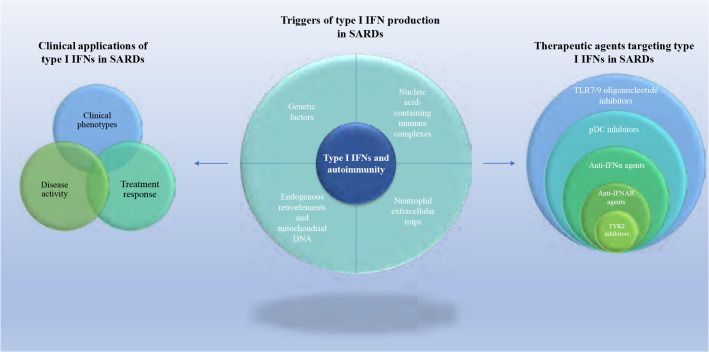
Type I IFN system dysregulation in systemic autoimmune rheumatic diseases. SARDs: Systemic autoimmune rheumatic diseases; IFNs: Interferons; IFNα: Interferon alpha; IFNAR: Interferon-alpha/beta receptor; TLR: Toll-like receptor; pDC: Plasmacytoid dendritic cell; TYK2: Tyrosine kinase 2.

Endogenous retroelements that are either nuclear DNA or mitochondrial DNA derived, are a potential source of endogenous nucleic acids with the ability to induce type I IFN production.^44^ Specifically, RNAs encoded by Alu retroelements, members of the short interspersed nuclear element repetitive element family, can increase the permeability of mitochondrial pores, enabling the release of mitochondrial DNA into the cytoplasm, activating cGAS and thereby inducing STING-dependent IFNβ production. This leakage of mitochondrial DNA into the cytosol triggers the activation of the noncanonical NOD-like receptor protein 3 (NLRP3) inflammasome pathway, contributing to inflammation-mediated tissue damage.^45^ Significantly, Ro60, a frequently targeted antigen in SLE and SjD, binds to Alu RNA, which is present in SLE ICs, while Ro60 deletion leads to elevated Alu and ISGs expression, indicating a regulatory function for Ro60.^46^ Furthermore, overexpression of long interspersed nuclear elements (LINE-1), a different family of retroelements, due to hypomethylation of several CpG elements in the 5’ regulatory region of LINE-1, has been observed in kidney and minor salivary glands (MSG) biopsies derived from lupus nephritis and SjD patients respectively.^47,48^ Notably, LINE-1 RNA expression significantly correlated with IFNα transcripts, while in vitro transcribed LINE-1 RNA induced expression of type I IFN mRNA. Additionally, this induction of type I IFN by in vitro–-transcribed LINE-1 RNA was suppressed by a TBK1 inhibitor, indicating the potential involvement of RNA sensors and MAVS.^47^ It is also possible that LINE-1 RNA might facilitate the release of mitochondrial DNA to the cytosol, activating the cGAS pathway.^44^ Apart from retroelements, a recent study in SLE further highlighted the significance of mitochondrial DNA in stimulating the cGAS pathway.^49^ Specifically, Caielli et al. showed that a defect in autophagic mitochondrial removal leads to the accumulation of mature red blood cells carrying mitochondria that undergo antibody-mediated internalisation by macrophages and induce type I IFN production through activation of the cGAS/STING pathway (**Figure 3**).^49^

## TYPE I IFNS AND SYSTEMIC AUTOIMMUNE RHEUMATIC DISEASES

### Systemic Lupus Erythematosus

The hypothesis regarding the potential pathogenic role of type I IFNs in SLE was first introduced in 1969 through a pivotal study by Steinberg et al. In this study, the administration of polyinosinic: polycytidylic acid, an inducer of type I IFNs, in the murine lupus model resulted in acceleration of the disease.^50^ A few years later, Skurkovich et al., followed by Hooks et al,. observed increased levels of type I IFN in the serum of SLE patients.^51,52^ Afterwards, Rich and colleagues demonstrated that recombinant IFNα could induce the formation of lupus inclusions, intracellular microtubular structures that were previously observed in glomerular endothelial cells of SLE and dermatomyositis (DM) patients.^53,54^ Since then, numerous studies have further elucidated the pathogenic role of type I IFNs in both murine models of lupus and SLE patients.^55,56^ It is now well established that up to 80% of SLE patients exhibit an overexpression of type I IFN-related genes in PBMCs with around 50% manifesting persistent increased type I IFN levels, detectable in plasma or serum.^57,58^ Apart from peripheral blood, the presence of type I IFN signature has also been identified in affected tissues of SLE patients, including the skin, joints, kidneys, and central nervous system (CNS), reinforcing the role of type I IFNs in tissue pathology.^59,60^ In a recent study, SLE patients with elevated baseline type I IFN activity had increased disease severity both at the initiation of the study and longitudinally, accompanied by an increased frequency of disease flares and an increased need for supplementary immunosuppressive agents.^61^ However, it’s crucial to note that the association between type I IFN activity and disease activity remains a subject of debate, as many longitudinal studies have failed to establish that type I IFN levels fluctuate predictably with changes in SLE disease activity.^62^ Increased type I IFN activity has further been linked to distinct clinical and serological features of SLE, particularly lupus nephritis, cutaneous manifestations (e.g. malar rash, alopecia) and the presence of anti-Sjögren’s-syndrome-related antigen A (anti-Ro/SSA), anti-Smith (anti-Sm), antiribonucleoprotein antibodies (anti-RNP), and anti-dsDNA antibodies (**Table 1**).^44^ However, it remains unclear whether the observed association between type I IFN activity and cutaneous and renal disease is primary or secondary, possibly stemming from an association between type I IFNs and SLE autoantibodies.^92^ Regarding the latter, several studies suggest that autoantibody immune complexes can directly stimulate type I IFN production.^38^ In a recent report, we have demonstrated type I IFN transcripts were elevated in renal tissues from patients with proliferative classes III/IV of lupus nephritis in association with impaired renal function.^67^ In the same context, another study showed an increased prevalence of lupus nephritis class III/IV in patients with higher activity of type I IFN, while in multivariate regression analysis, type I IFN signature has been revealed as a stronger predictor of class III/IV nephritis than complement C3 levels or anti-dsDNA antibodies.^68^ Apart from renal and skin disease, almost all clinical features, including pulmonary, musculoskeletal, CNS, vascular, and hematologic manifestations have been associated with increased type I IFN activity; however, the exact pathogenetic role of type I IFNs in the context of these manifestations has not been elucidated yet.^93^ Furthermore, in several studies SLE patients have active disease affecting multiple organ systems, potentially confounding the conclusions drawn due to the presence of co-existing manifestations, particularly when the comparator group includes healthy individuals.^94^ Lastly, specific clinical and serologic features are linked to different IFN subtypes. For example, high IFN-α levels were associated with mucocutaneous manifestations, anti-Ro52 and anti-La antibodies while elevated IFN-γ levels were coupled to arthritis, nephritis, and anti-Ro60 antibodies.^95^

**Table 1.** Clinical, laboratory, and therapeutical implications of type I IFN activity in systemic autoimmune rheumatic diseases.

**Disease**	**Type I IFN activity**	**Sample**	**Association**	**Reference**
SLE	Increased	Serum, peripheral blood	Increased disease activity	^61,63–65^
		Periphreal blood, skin biopsy, kidney biopsy	Musculoskeletal, cutaneous disease, class III/IV lupus nephritis	^66–69^
		Peripheral blood leukocytes and monocytes	Anti-Sm, anti-RNP, anti-Ro/SSA and anti-dsDNA	^58,64^
		Peripheral blood	Better response to anifrolumab	^70^
	Decreased	Peripheral blood	Better response to rontalizumab	^71^
RA	Increased	Peripheral blood	Decreased disease activity	^72^
		Serum	Increased disease activity	^73^
		Peripheral blood, serum	Arthritis development, cardiovascular events	^73,74^
		Peripheral blood	ACPA and anti-CarP	^75^
		Peripheral blood	Non-response to rituximab	^76^
	Increased IFNβ/IFNα activity ratio	Serum	Better response to TNF inhibition	^77^
			Non-response to TNF inhibition	^78^
SjD	Increased	Peripheral blood monocytes	Increased disease activity	^79^
		Peripheral blood monocytes, MSG biopsy	Extraglandular manifestations, lymphoma	^80,81^
		Peripheral blood monocytes	Anti-Ro/SSA and anti-La/SSB	^79^
		Peripheral blood monocytes	Increased effect of belimumab on immunoglobulin production	^82^
	Increased IFNγ/IFNα mRNA ratio	Serum	Non-response to TNF inhibition	^83^
		MSG biopsy	Lymphoma	^84^
SSc	Increased	Serum	Muscle, kidney, cardiac, lung disease	^85,86^
		Peripheral blood, serum	Anti-Scl-70, anti-RNP and anti-Ro/SSA	^87,88^
DM and JDM	Increased	Peripheral blood	Increased disease activity	^89,90^
		Peripheral blood monocytes	Increased risk for requiring treatment intensification	^91^

SLE: Systemic lupus erythematosus; RA: Rheumatoid arthritis; SjD: Sjögren’s disease; SSc: Systemic sclerosis; DM: Dermatomyositis; JDM: Juvenile dermatomyositis; IFN: Interferon; IRF4: Interferon regulatory factor 4; IFIT1: IFN-induced protein with tetratricopeptide repeats 1; ISG-15: Interferon stimulated gene 15; IP-10: Interferon-inducible protein-10; MSG: Minor salivary gland; anti-Sm: Anti-Smith autoantibodies; anti-RNP: Antiribonucleoprotein antibodies; anti-Ro/SSA: Anti-Sjögren’s-syndrome-related antigen A autoantibodies; anti-dsDNA: Anti-double stranded DNA autoantibodies; anti-La/SSB: Anti-Sjögren’s-syndrome-related antigen B autoantibodies; anti-Scl-70: Anti-topoisomerase I antibodies; TNF: Tumour necrosis factor.

The pivotal role of type I IFNs in the pathogenesis of SLE has prompted the development of several biologics targeting this pathway. First rontalizumab, a humanised IgG1 anti-IFNα monoclonal antibody (mAb) failed to achieve its primary endpoint (BILAG-based Composite Lupus Assessment (BICLA) responses) in a phase II trial and the development of this drug was discontinued.^71^ Sifalimumab, a fully humanised IgG1 anti-IFNα mAb, and AGS-009, a humanised IgG4 anti-IFNα mAb, were evaluated in early-phase clinical trials, but their development was also discontinued despite promising initial results regarding safety and efficacy.^96,97^ The IFNα kinoid, an immunotherapeutic vaccine leading to the development of anti-IFN neutralising antibodies, did not meet its primary endpoint of BICLA response rate although it demonstrated improvements in clinically relevant secondary outcomes.^98^ JNJ-55920839, a mAb against IFNα and IFNω, was well tolerated in a phase I trial; however additional studies are warranted to further explore safety and efficacy.^99^ Anifrolumab is a mAb against IFNAR1, thereby inhibiting the activity of all type I IFNs.^94^ In three large double-blinded randomised controlled trials (RCTs), namely MUSE, TULIP-1, and TULIP-2 trials, anifrolumab has demonstrated superiority over placebo in decreasing disease activity, glucocorticoid dosage, and the severity of cutaneous manifestations in SLE.^100^ Notably, a recent post-hoc analysis of combined data from phase III trials showed that SLE subjects displaying increased baseline type I IFN signature experienced more significant improvement after anifrolumab therapy compared to those with a low type I IFN signature.^101^ Moreover, a recently published phase 2 trial for the use of anifrolumab in active lupus nephritis patients did not meet its primary endpoint. Nevertheless, a greater number of patients in the anifrolumab group achieved complete renal response compared to the placebo group.^102^ An ongoing phase 3 clinical trial is currently testing anifrolumab for lupus nephritis (NCT05138133). Furthermore, TYK2 inhibitor deucravacitinib demonstrated superiority over placebo in reducing disease activity across various measures in a phase II trial.^103^ Brepocitinib, an inhibitor targeting both JAK1 and TYK2, is currently under investigation in a phase II clinical trial (NCT03845517).^104^ Lastly, litifilimab, a humanised IgG1 against blood dendritic cell antigen 2 (BDCA2) receptor on pDCs, was tested in a phase II trial demonstrating improvements in inflamed joints and skin manifestations (**Table 2**).^105^

**Table 2.** Completed clinical trials with agents targeting the type I interferon pathway in systemic autoimmune rheumatic diseases.

**Agents targeting type I IFN system**		**Completed clinical trials**					
**Mechanism of action**	**Drug name**	**Disease**	**Clinical trial phase**	**Primary outcome measures**	**Clinical Trial Registration number**
Anti-IFNα	Rontalizumab	SLE	Phase II	BILAG score at week 24 The primary outcome was not met. No significant difference between the placebo and rontalizumab groups.	NCT00962832
	Sifalimumab	SLE	Phase IIb	SRI-4 response at day 365 The number of patients achieving the primary outcome was greater for sifalimumab versus the placebo group.	NCT01283139
			Phase IIa	TEAEs Unpublished results.	NCT00657189
		DM	Phase I	TEAEs The small safety database size limited the interpretation of the safety profile. TEAEs were of low severity and TESAEs were uncommon.	NCT00533091
	Interferon-α-kinoid	SLE	Phase I/II	TEAEs IFN-α-kinoid was well tolerated. Most TEAEs were of mild/moderate severity. 1 TESAEs (SLE flare)	NCT01058343
	AGS-009	SLE	Phase I	TEAEs AGS-009 was safe and well tolerated at all dose levels with no TESAEs.	NCT00960362
	JNJ-55920839	SLE	Phase I	TEAEs JNJ-55920839 was well tolerated. Higher percentage of infections in the JNJ-55920839 group. 2 TESAEs (both cases of herpes zoster infection)	NCT02609789
Anti-IFNAR	Anifrolumab	SLE (FDA approved)	Phase IIb	SRI-4 response at 6 months The primary outcome was met by more patients in the anifrolumab versus placebo group with greater effect size in patients with a high IFN signature at baseline.	NCT01438489
			Phase III	SRI-4 response at week 52 The primary outcome was not met. No significant difference in SRI-4 response between the placebo and anifrolumab group. However, patients in the anifrolumab group had improved CLASI and BICLA responses.	NCT02446912
			Phase III	BICLA response at week 52 The primary outcome was met by more patients in the anifrolumab versus placebo group.	NCT02446899
		SSc	Phase I	TEAEs Adequate safety and tolerability profile. Most TEAEs were of mild/moderate severity. Of 4 TESAEs, only CML was considered possibly treatment-related.	NCT00930683
TYK2 inhibition	Deucravacitinib	SLE	Phase II	SRI-4 response at week 32 The primary outcome was met by more patients in the deucravacitinib versus placebo group	NCT03252587
JAK1/TYK2 inhibition	Brepocitinib (PF-06700841)	SLE	Phase II	SRI-4 response at week 52 Unpublished results.	NCT03845517
pDC depletion	Litifilimab (anti-BDCA2 mAb)	SLE	Phase II	Change from baseline in 28 Joint Count at week 24 Patients who received litifilimab achieved a greater reduction in the number of tender and swollen joints compared to placebo.	NCT02847598
			Phase I	TEAEs Litifilimab was safe and well tolerated at all dose levels. Most TEAEs were of mild/moderate severity.	NCT02106897
	VIB7734 (anti-ILT7)	SLE	Phase II	BICLA response and oral glucocorticoid reduction response at week 48 The primary outcome was not met. No significant difference between the placebo and the VIB7734 group	NCT04925934
		SLE, SjD, SSc, DM	Phase I	TEAEs Acceptable safety profile with no TESAEs	NCT03817424
RNA degradation	RSLV-132	SLE	Phase II	Change from baseline in CLASI activity scores at day 85 and 169 The primary outcome was not met. No significant difference in the mean change in CLASI score between the RSLV-132 and placebo group at either time point.	NCT02660944
			Phase I	TEAEs RSLV-132 was well tolerated with a favorable safety profile. Most TEAEs were of mild severity	NCT02194400
		SjD	Phase II	Change from baseline in IFN-inducible genes expression at Day 99 Increased expression of IFN-inducible genes in the RSLV-132 versus placebo group.	NCT03247686

SLE: Systemic lupus erythematosus; SjD: Sjögren’s disease; SSc: Systemic sclerosis; DM: Dermatomyositis; CML: Chronic myelogenous leukemia; IFN: Interferon; IFNα: Interferon alpha; IFNAR: Interferon-alpha/beta receptor; JAK: Janus kinase; TYK2: Tyrosine kinase 2; pDC: Plasmacytoid dendritic cell; BDCA2: Blood dendritic cell antigen 2; ILT7: immunoglobulin-like transcript 7; FDA: Food and Drug Administration; BILAG: British Isles Lupus Assessment Group; SRI-4: Systemic lupus erythematosus responder index 4; TEAEs: Treatment-emergent adverse events; TESAEs: Treatment-emergent serious adverse events; BICLA: British Isles lupus assessment group based composite lupus assessment; CLASI: Cutaneous Lupus Erythematosus Disease Area and Severity Index.

### Rheumatoid Arthritis

The presence of peripheral blood type I IFN signature is observed in approximately half of the patients with RA.^106^ Notably, this type I IFN signature can discriminate patients with self-limiting arthritis from those that progress to established RA.^107^ In addition, increased type I IFN-inducible gene expression is associated with elevated anti-citrullinated protein antibodies (anti-ACPA) titers, more destructive/erosive arthritis, and persistent inflammation.^74,106^ Type I IFN signature is also linked to nonresponse to rituximab ^76^, while several studies have identified pre-treatment serum ratio of IFNβ to IFNα as a predictor of treatment response to TNF inhibitors in RA.^77,78^ A recent metanalysis, highlighted that the correlation between the activation of the type I IFN pathway and the clinical response to anti-TNF treatment varied in studies utilising different assays, biosamples, and sample timings.^108^ Multiple studies have confirmed the presence of pDCs in synovial tissue, along with an elevation in IFNα and IFNβ levels in the synovial fluid.^109,110^ Interestingly, stimulation of TLR3/TLR7 in pDCs located primarily in the synovium, can induce IFNα production which, in turn, enhances TLR4-mediated signaling leading to increased expression of proinflammatory cytokines including IL1b and IL18.^111^ Conversely, stimulation of chondrocytes and synovial fibroblasts with IFNβ can increase production of IL1 receptor antagonist, suggesting an anti-inflammatory effect for IFNβ.^112^ However, subcutaneous administration of IFNβ in RA patients did not result in disease improvement in a phase II clinical trial.^113^

A phase II study evaluating the efficacy and safety of anifrolumab in patients with RA and a high type I IFN signature was prematurely stopped due to recruitment difficulties. Results from this trial showed that the safety profile of anifrolumab was similar to already published trials in SLE; however, no conclusions regarding clinical efficacy could be drawn due to the limited number of patients who completed this trial.^114^

### Sjögren’s disease

The presence of type I IFN signature has been detected in peripheral blood, PBMCs, isolated monocytes, B cells, minor salivary glands (MSGs), and ocular epithelial cells of SjD patients.^115^ The detection of infiltrating pDCs in MSGs of patients with SjD, strongly suggests a potential role of IFNα production by these cells within the glandular microenvironment.^116^ Similarly, an RNA-sequencing analysis showed that pDCs derived from patients with SjD exhibit elevated expression of IFN-related genes and secrete higher levels of IFNα and IFNβ in comparison to pDCs derived from non-SjD patients.^117^ A recent study showed that type 2 conventional dendritic cells (cDC2s) from patients with SjD have impaired antigen uptake and processing, including self-antigens from MSG epithelial cells, while those changes are strongly linked to anti–SSA positivity and the presence of elevated type I IFNs.^118^ Furthermore, type I IFN activation in neutrophils from SjD patients can lead to mitochondrial damage and increased reactive oxygen species production with subsequent increased generation of NETs, indicating a potential role for NETs in type I IFN dysregulation associated with SjD.^119^ Increased type I IFN-inducible gene expression is associated with higher clinical disease activity together with higher B cell activating factor (BAFF) expression and increased autoantibody production.^79^ Notably, we have previously shown that treatment with TNF inhibitor etanercept can exacerbate IFNα and BAFF overexpression, suggesting a potential mechanism for the lack of efficacy of this therapeutic agent in SjD.^83^ In addition, patients with SjD and systemic extra-glandular involvement exhibit elevated expression of type I IFN-related genes compared to patients with a disease limited to glandular features.^80^ Apart from type I IFN signature, type II IFN-related genes can be overexpressed in MSG biopsy samples from SjD patients and IFNγ/IFNα ratio may serve as a biomarker for the diagnosis of SjD-related lymphoma.^84^ In the context of SjD-related lymphomagenesis, Cinoku et al. showed that expression of ISG-15, a type I IFN-inducible gene, is increased in both MSGs biopsy samples and peripheral blood from patients with SjD-related lymphoma, representing a novel biomarker for lymphoma development among SjD patients.^81^

A low dose of orally administered IFNα improved salivary output and decreased complaints of xerostomia in a phase II clinical trial of patients with SjD^120^; nevertheless, combined results from two phase III trials failed to confirm this finding.^121^ Endogenous RNA in association with ICs can be a potential triggering factor for type I IFN production. In this context, RSLV-132, an RNase fused to human IgG1 Fc domain with the ability to degrade circulating immunostimulatory RNAs and therefore inhibit production of type I IFNs, was evaluated in a phase II trial. RSLV-132 did show clinically meaningful improvements, primarily regarding severe fatigue in patients with SjD.^122^ Additionally, trials assessing anifrolumab (NCT05383677) and deucravacitinib (NCT05946941) for SjD are currently in the recruitment phase.

### Systemic Sclerosis

Increased expression of type I IFN-related genes has been detected in peripheral blood and PBMCs of SSc patients.^123^ Accumulation of endogenous DNA damage has been recognised as a significant pathogenic mechanism in multiple SARDs.^3^ In this context, Vlachogiannis et al. showed that DNA damage in SSc PBMCs strongly correlates with type I IFN-inducible genes’ expression.^124^ However, whether DNA damage precedes/induces type I IFN upregulation or if chronic type I IFN activation leads to increased DNA damage and dysregulation of DNA repair mechanisms remains largely unclear.^125^Affected tissues of SSc patients such as the skin and lungs also exhibit increased type I IFN activity.^126,127^ Regarding autoantibody production in SSc, the presence of type I IFN signature is associated with the presence of anti-topoisomerase antibodies and anti–U1 ribonucleoprotein (U1RNP) antibodies while negatively correlates with anti-RNA polymerase III antibodies.^87,128^ In addition, SSc patients with anti-SSA and anti-U1RNP antibodies are more likely to have increased levels of type I IFN compared to their seronegative counterparts.^88^ Type I IFN signature is also associated with more severe cutaneous, vascular, pulmonary, and muscular manifestations.^85,86^ In the same context, type I IFN-inducible cytokines are found to predict skin, lung, vascular, and gastrointestinal progression in patients with limited cutaneous SSc.^123^ Notably, Assassi et al. showed that an increased type I IFN score in SSc-related interstitial lung disease can serve as a predictor for better response to immunosuppressive treatment, suggesting its potential utility in identifying patients who may derive the most benefit from mycophenolate mophetil or cyclophosphamide.^129^ Lande et al. showed that CXCL4 is capable of organising microbial and self-DNA into complexes that can induce TLR9-mediated IFNα production in pDCs of patients with SSc. Interestingly, CXCL4-DNA complexes were detected in vivo, both in circulation and skin tissues of SSc patients, and correlated with type I IFN levels.^40^ Towards the same direction, another study showed that infiltration of SSc skin by CXCL4/IFNα-producing pDCs can exacerbate skin fibrosis in a mouse model of SSc.^130^ Furthermore, anti-CXCL4 antibodies were detected in approximately half of SSc patients, positively correlating with serum IFNα levels.^131^ Indeed, further work showed that anti-CXCL4 antibodies can be present in patients with very early diagnosis of SSc, indicating that this mechanism may play a role very early in the disease’s pathogenesis.^132^

An early phase I trial of SSc patients showed that anifrolumab was well tolerated and achieved peak type I IFN inhibition in whole blood and skin within 1 and 7 days, respectively.^133^ A follow-up study demonstrated that anifrolumab administration can significantly downregulate T cell-associated proteins and upregulate type III collagen degradation marker, suppressing T cell activation and collagen accumulation.^134^

### Dermatomyositis

Upregulation of type I IFN genes has been observed both in blood and in affected tissues including the skin and muscle of patients with adult and juvenile DM.^128^ The type I IFN signature also appears to correlate with disease activity in both adult and juvenile DM.^89,90^ Towards the same direction, a recent study showed that overexpression of Siglec-1, a type I IFN-related gene, is associated with clinical disease activity and suboptimal treatment response in patients with juvenile DM.^91^ Moreover, patients with DM have markedly higher expression of type I IFN-related genes compared to patients with immune-mediated necrotising myopathy and inclusion body myositis.^135^ In the same context, Ekholm et al. documented an association between the type I IFN signature and a subgroup of myositis patients with autoantibodies against RNA-binding proteins, highlighting that different molecular mechanisms may predominate in different subgroups of myositis.^136^ In addition, infiltration of pDCs, a potential local source of IFNα, has been observed in muscle and skin biopsies derived from patients with DM.^137^ Apart from IFNα, expression of IFNβ is also increased and positively correlates with blood type I IFN signature in DM patients.^138^ This is further supported by the fact that IFNβ treatment in patients with multiple sclerosis can induce severe DM.^139^ Interestingly, a recent study demonstrated that high concentrations of IFNβ can decrease muscle stem cell proliferation in vitro, leading to muscle repair deficit in DM.^140^ It is also worth mentioning that, MDA5^+^ DM patients have a significantly higher type I IFN signature in the skin and blood, while MDA5- DM patients exhibit a stronger signature in the muscle.^141^ Moreover, increased expression of IFNκ by keratinocytes has been observed in the skin of patients with MDA5+ DM.^142^ Lastly, a study aiming to elucidate the association between distinct clinical phenotypes of inflammatory myopathies with the presence of serum MSAs or MAAs, as well as with type I IFN activation is currently ongoing.^143^ In a phase 1b clinical trial, sifalimumab, an anti-IFNα mAb, suppressed T cell-related proteins and type I IFN activation, while also leading to clinical improvements in DM and PM patients.^144^ A trial testing brepocitinib in adult DM patients is currently in the recruitment phase (NCT05437263).

## CONCLUSIONS

Dysregulation of type I IFN responses is greatly involved in the development of systemic autoimmunity. Aberrant functionality of type I IFN-secreting cells and genetic variations affecting type I IFN production, regulation, and downstream signaling, in combination with epigenetic alterations can lead to the breakdown of immune tolerance and subsequent development of autoimmune disorders. However, the exact mechanisms through which alterations in distinct parts of the type I IFN system contribute to the pathogenesis of different SARDs are not yet fully elucidated. The diversity in genetic and environmental backgrounds, pathophysiological mechanisms, and ultimately clinical phenotypes among these diseases adds complexity to the analysis and interpretation of research findings. In this context, future research may focus on identifying specific molecular dysregulation in IFN pathways that differ among distinct clinical phenotypes, laboratory features, and different levels of disease severity. From a therapeutic perspective, targeting the type I IFN system with the goal of ameliorating immunopathology seems an appealing and promising approach for the treatment of SARDs. The inconsistent results observed thus far in implementing this therapeutic strategy for SARDs highlight the necessity to identify clinical and molecular phenotypes that would derive the most benefit from such interventions and patient groups at risk of experiencing adverse events during anti-IFN therapy. Therefore, the examination of individual samples from clinical trials along with patient subgrouping based on molecular phenotypes is of crucial importance to significantly impact and individualise therapeutic approaches in SARDs.
